# Is timing of reproduction according to temperature sums an optimal strategy?

**DOI:** 10.1002/ece3.5601

**Published:** 2019-10-02

**Authors:** Jacob Johansson, Kjell Bolmgren

**Affiliations:** ^1^ Department of Biology Theoretical Population Ecology and Evolution Group Lund University Lund Sweden; ^2^ Swedish National Phenology Network Unit for Field‐based Forest Research c/o Swedish University of Agricultural Sciences Lammhult Sweden

**Keywords:** annual plants, climate change, phenology, temperature sums, timing of reproduction

## Abstract

Temperature sums are widely used to predict the seasonal timing of yearly recurring biological events, such as flowering, budburst, and hatching. We use a classic energy allocation model for annual plants to compare a strategy for reproductive timing that follows a temperature sum rule (TSR) with a strategy that follows an optimal control rule (OCR) maximizing reproductive output. We show that the OCR corresponds to a certain TSR regardless of how temperature is distributed over the growing season as long as the total temperature sum over the whole growing season is constant between years. We discuss such scenarios, thus outlining under which type of variable growth conditions TSR maximizes reproductive output and should be favored by natural selection. By providing an ultimate explanation for a well‐documented empirical pattern this finding enhances the credibility of temperature sums as predictors of the timing of biological events. However, TSR and OCR respond in opposite directions when the total yearly temperature sum changes between years, representing, for example, variation in the length of the growing season. Our findings have implications for predicting optimal responses of organisms to climatic changes and suggest under which conditions natural selection should favor photoperiod versus temperature control.

## Graphical abstract

Temperature sums are widely used to predict the seasonal timing of yearly recurring biological events. By formulating a temperature‐sum rule in mathematical terms and contrasting it with predictions from a classic plant energy‐allocation model we link a well‐established empirical pattern with traditional optimal‐control modeling and life‐history theory.

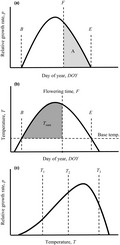

## 1 INTRODUCTION

Temperature sums have been used to predict the seasonal timing of biological events at least since the 18th century (Abbe, 1905; Réaumur, 1735). They are widely applied within agriculture and ecology and have been used to predict, for example, harvest dates, appearance of pest insects, budburst in trees and flowering (Bonhomme, 2000; Wilson & Barnett, 1983). The last decades have seen a renewed interest of using temperature sums for predicting phenological events in a changing climate (Murray, Cannell, & Smith, 1989; Olsson, Bolmgren, Lindström, & Jönsson, 2013).

From a physiological point of view, it is not surprising that there is a correlation between temperature sums and timing of biological events (Brown, Gillooly, Allen, Savage, & West, 2004). Temperature increases the speed of chemical reactions and thus tends to increase growth and development rates (Thompson, 1942). It is often argued; however, that “nothing in ecology make sense except in the light of evolution” (Dobzhansky, 1973). Indeed, organisms have evolved other strategies to control the timing of their activities and may rely on other cues than temperature including photoperiod, soil moisture, and food abundance. We therefore here seek an ultimate explanation for why organisms should time their activities according to temperature sums. We focus on timing of reproduction for annual plants. This is motivated by annual plants being an important group for which temperature sums are applied, including many crop species and weeds (Bonhomme, 2000; Chauhan, Ryan, Chandra, & Sadras, 2019; Moore & Remais, 2014). The model, we develop is, however, of a general character and has relevance also for other organisms, such as annual social insects (e.g., Macevicz & Oster, 1976; Mitesser, Weissel, Strohm, & Poethke, 2007).

We consider growing conditions that undergo seasonal changes and furthermore differ between years. We ask under which circumstances timing of reproduction according to a temperature sum is an expected evolutionary outcome by being the strategy that maximizes reproductive output (King & Roughgarden, 1983; Stearns, 1992 but see Metz, Mylius, & Diekmann, 2008). To do this, we here compare timing of reproduction for plants following temperature sum rules to the optimal timing of reproduction in a classic energy allocation model.

According to Bonhomme (2000), phenological predictions based on temperature sums critically rest on the assumptions that (a) developmental rates depend linearly on temperature assumptions and that (b) no factor apart from temperature limit development. To accord with these assumptions, we will here use a plant growth model which assumes that the relative growth rate depends linearly on temperature and no other limiting factors.

In order to shed light on the generality of our findings, and as a robustness test, we shall also compare our results with two additional versions of the plant model, which have been used to study the effects of limiting factors (see key assumption ii above) such as herbivory and self‐shading on optimal reproductive phenology. This comparison is presented in Appendix 1. Effects of nonlinear relationships between temperature and development rates (see key assumption i above) are widely acknowledged and often discussed as a limitation of temperature sum models and covered elsewhere (see e.g., Moore et al., 2014).

## 2 METHODS

### 2.1 Growth and reproduction model

**Figure 1:**
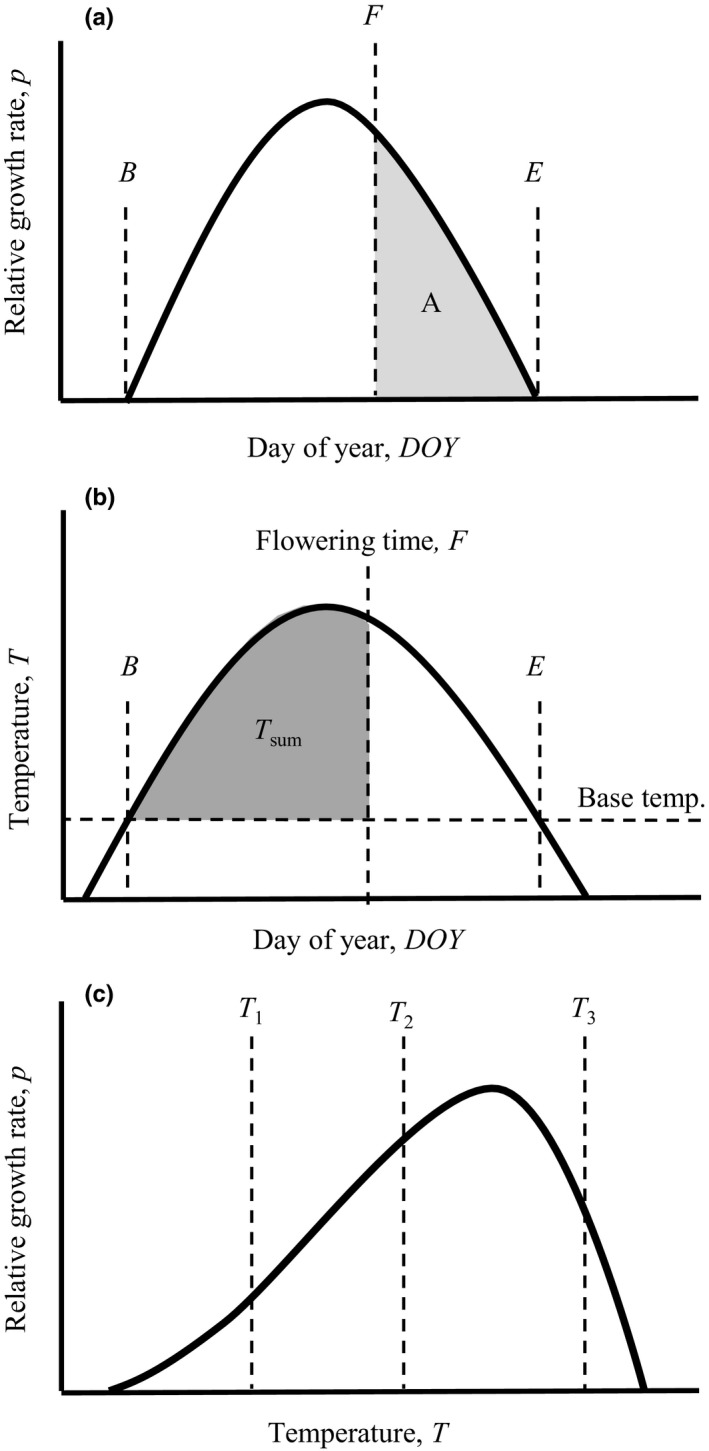
Relationships between flowering time, relative growth rates, and temperature sums. Relative growth rate, *p*, undergoes seasonal variation (a) and according to the model, flowering time *F* is optimal when the integral *A* is equal to 1 (Equation 3). The sum of daily temperatures above a certain baseline value is often used to predict the timing of biological events. In analogy, we here envision that the integral of the temperature curve above the baseline (*T*_sum_) is used to predict flowering time as shown in (b). The beginning (*B*) and end (*E*) of the growing season occur when the temperatures exceed and falls below the base temperature, respectively. As shown in (c) growth rates typically increase with temperature up to a certain point and then decrease at high temperatures. Following a common practice, we assume a linear relationship between relative growth rate and temperature (such as between *T*_1_ and *T*_2_ but not between *T*_2_ and *T*_3_)

We consider the growth and energy allocation model for annual plants by Paltridge and Denholm (1974) that in turn is closely related to models by Cohen (1971, 1976) and further analogous to a model of annual social insects (Macevicz & Oster, 1976). Produced biomass is partitioned between vegetative mass *V* and reproductive mass *R* according to a time‐dependent control function *u*(*t*), where *u* represents the relative investment (0 ≤ *u* ≤ 1) into vegetative growths. Relative growth rate of a plant, *p*(*t*), undergoes seasonal variation and the growing season begins day *B* and ends day *E* (Figure 1a). The model assumes that the vegetative part grows according to:

(1) $$dV/dt=u\left(t\right)p\left(t\right)V\left(t\right)\;\text{with}\;V\left(B\right)=V_{0}.$$

and that the reproductive part grows according to:

(2) $$dR/dt=\left(1-u\left(t\right)\right)p\left(t\right)V\left(t\right)\;\text{with}\;R\left(B\right)=0.$$

Previous analyses of this model show that a single switch (bang‐bang control) from pure investment into vegetative growth (*u* = 1) to pure investment into reproductive growth (*u* = 0) is the optimal strategy (Paltridge & Denholm, 1974, see e.g., Intrilligator, 1971 for an introduction to optimal control theory). We interpret the timing of this switch as flowering time denoted *F*, by interpreting flowering as the initial stage of reproductive phase (cf. King & Roughgarden, 1983). The reproductive mass may, however, represent other organs as well, for example, fruits, seeds and necessary structures for their functionality.

The optimal choice of flowering time *F* balances the contrasting goals of growing large (by switching as late as possible) and spending a long time investing into reproductive biomass (by switching as early as possible). The optimal flowering time occurs specifically when (King & Roughgarden, 1983; Shitaka & Hirose, 1993):

(3) $$\int_{F}^{E}p\left(t\right)dt=1$$

(i.e., when the area *A* in Figure 1a is equal to 1). This result can be intuitively understood by considering that the integral in Equation 3 represents the contribution of one unit of vegetative biomass contributes to the final reproductive output from the switch point *F* to the end of the season *E* (see e.g., Lindh, Ripa, & Johansson, 2018; Poitrineau, Mitesser, & Poethke, 2009). Early in the season (when *F*–*E* is large), the integral is above one, and hence investment into vegetative mass contributes more to the final reproductive output than direct reproductive investments. Late in the season (when *F*–*E* is small), on the other hand, the integral is below one, and the plant should therefore exclusively invest into reproduction. The implications of Equation 3 for adaptation of flowering times to long‐term climate changes, where the curve *p* takes on a new shape compared to a fixed, historic shape (akin to a press perturbation in e.g., Bender, Case, & Gilpin, 1984) are discussed in Johansson, Bolmgren, and Jonzén (2013) but in this study we will instead consider that that *p* undergoes interannual fluctuations.

### 2.2 Temperature‐sum phenology model

The typical temperature sum model can be formulated as (Wang, 1960):

(4) $$\sum_{B}^{F}T_{t}=T_{\text{sum}}$$

where *T_t_* is the excess temperature above some base temperature day *t* (Figure 1a) and *T*_sum_ is the temperature sum which coincides with a particular biological event (in our case the flowering time *F*). We consider a scenario where the temperature exceeds the base temperature between days *B* and *E*, corresponding to the beginning and end of the growing season. To shorten notation, we refer to *T_t_* simply as “temperature” from hereon. In order to compare Equations 3 and 4, we then transform the latter in two steps. First, we treat the temperature sum as an integral of a continuous temperature curve *T*(*t*) over an interval of time, instead of a sum over a discrete number of days. Second, we assume that there is a positive linear relationship between the relative growth rate *p* and temperature (cf. the interval between *T*_1_ and *T*_2_ in Figure 1c), which is a general prerequisite for temperature sums model to be accurate (Bonhomme, 2000). Accordingly, we assume *p*(*t*) = *kT*(*t*), where *k* is a proportionality constant, and that the base temperature is subtracted from *T*(*t*). We then express the temperature sum in terms of productivity as *P* = *kT*_sum_ and obtain (Figure 1a):

(5) $$\int_{B}^{F}p\left(t\right)dt=P$$

Note that the expressions in Equations 4 and 5 thus have the same meaning, only that the expression in Equation 5 is adapted so that it can be analyzed within the plant growth model we consider here (Equations 1 and 2).

### 2.3 Flowering time strategies and interannual variation in growing conditions

To keep notation short, a plant with timing of flowering according to Equation 3 is here said to follow an optimal control rule (OCR) and a plant that flowers according to Equation 5 is said to follow a temperature sum rule (TSR). We will assume that the relative growth rates *p*(*t*) undergoes interannual fluctuations driven by underlying fluctuations in temperatures (*T*(*t*)). As a consequence, also the OCR and TSR will vary between years.

If the environment undergoes interannual fluctuations, the optimal energy allocation strategy may not always be of bang‐bang type as we assume here and this requires us to make some further assumptions and clarifications regarding the OCR. King and Roughgarden (1982) showed that mixed investments into vegetative and reproductive biomass may be an optimal strategy when the length of the season varies across years (see also Mitesser et al., 2007). The model by King and Roughgarden (1982) considered; however, that *u*(*t*) was fixed between years which implies that bang‐bang strategies may not reach the reproductive stage if the season is too short. Here we instead consider that the optimal strategy is flexible and adjusts its allocation schedule to the growth conditions of each specific year. Our assumption that the optimal allocation strategy is of a bang‐bang type is further motivated by the fact that this is the fitness‐maximizing strategy for any specific *p*(*t*). It may be unlikely that such a strategy may result from evolution, especially because the optimal choice of switching to reproduction requires information of the environmental conditions after the switch point (Equation 3) and “perfect knowledge” only exists in theory. The optimal strategy thus defined is nevertheless a useful reference point since it is independent of organism‐ or situation‐specific constraints affecting behavioral or selective responses to environmental variation (cf. Parker & Maynard‐Smith, 1990).

## 3 RESULTS

First, we see that the integral of the temperature curve is constant under both the temperature sum rule (Equation 5) and the optimal control rule (Equation 3). The interval limits differ; however, the temperature sum rule (TSR) integrates from the beginning of growth to flowering start (*T*_sum_ in Figure 1b) while the optimal control rule (OCR) integrates from flowering start to end of growth (Figure 1a). This shows that TSR and OCR from this model do not correspond to each other in general. In one specific case, however, they do coincide. This occurs^1^ when the integral of *p*(*t*) over whole growing season, henceforth denoted *P*_tot_, is constant between years, i.e.;

(6) $$\int_{B}^{E}p\left(t\right)dt=P_{\text{tot}}$$

and when furthermore TSR predict the optimal flowering time in a given year, i.e., when;

(7) $$P=P_{\text{tot}}-1$$

Under these conditions, *P*, which is fully controlled by *T*_sum_ (Figure 1a,b, Equations 3 and 5), is namely constant as well. Note also that a constant *P*_tot_ means the temperature sum accumulated over the whole growing season (from *B* to *E*) does not change between years.

**Figure 2:**
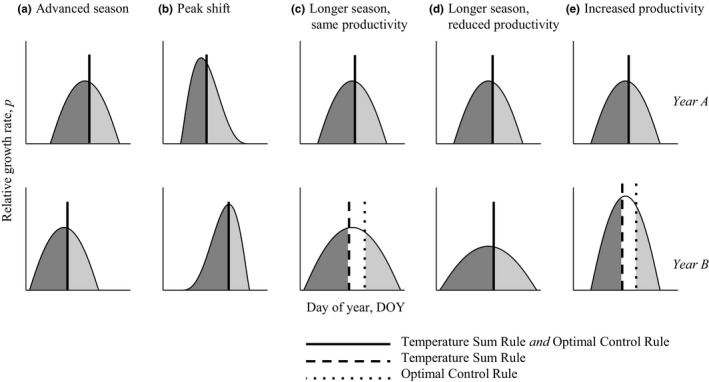
Effects of changes in growing conditions on strategies to control flowering time in five different scenarios (a–e). Growing conditions, here represented by the seasonal variation in the relative growth rate *p*, changes between two consecutive years denoted as A (top row) and B (bottom row). In all cases, it is assumed that the temperature sum rule (TSR) coincides with the optimal control rule (OCR) in year A. Depending on how the environment changes, OCR and TSR may coincide also in year B (solid, vertical lines) or show differential responses (dashed and dotted, vertical lines)

From these considerations, it follows that flowering times according to TSR and OCR coincide for any change in shape of *p*(*t*) as long as *P*_tot_ is constant. Such changes could, for example, include an advanced growing season (with preserved shape otherwise as in Figure 2a), shifted peak temperature day (Figure 2b) and if the season length varies at the same time as a longer season reduces productivity (Figure 2d). Hence, under those rather flexible conditions TSR will produce the optimal solution and should be favored by natural selection.

In contrast, TSR will not produce the same flowering time as the OCR when *P*_tot_ changes between years. When this happens a TSR predicts a change of the flowering time in the opposite direction compared to OCR: both under a longer growth season with the same productivity (Figure 2c) and a constant length of the growth season but with increased temperature (Figure 2e), TSR causes too early flowering compared to the optimal flowering time.

### 3.1 Effects of alternative model assumptions

We studied the robustness of our predictions above by comparing how TSR and OCR respond to variation in *p*(*t*) depends on different assumptions in the plant growth model. Specifically, we compared predictions of the basic growth model analyzed above (Equations 1 and 2) with a model version where the plant growth rates slow down as the plant grows larger, for example, due to self‐shading or competition among conspecifics growing densely together (see e.g., Lindh et al., 2016) and a model version representing plant biomass being lost due to herbivory or senescence (King & Roughgarden, 1983). Overall we find only subtle differences between the predictions of the different models. For example, when vegetative and reproductive biomass is lost at different rates, OCR and TSR are approximately but not exactly equal when *p*(*t*) varies but *P*_tot_ is fixed across years (cf. Figure 2a,b,d). It can also be noted that when vegetative and reproductive biomass is lost at the same rate, the optimal timing of reproduction is the same as in the basic growth model (e.g., Lindh et al., 2018). See Appendix 1 for details.

## 4 DISCUSSION

By formulating a temperature sum rule (TSR) in mathematical terms and contrasting it with a model plant obeying an optimal control rule (OCR) we link a well‐established empirical pattern with a long tradition of energy allocation modeling in studies of life history evolution.

Our analysis outlines general conditions under which TSR is an optimal strategy and therefore can be expected to be favored by natural selection. Specifically, we find that TSR is a robust, fitness‐maximizing strategy for in principle any variation of the distribution of temperatures over the growing season, as long as the total temperature sum over the growing season does not change between years. More generally, it is required that the total accumulated daily development rates are constant. This requirement may seem rather restrictive, but is not unlikely, especially if we take into account that other factors than temperature may constrain a plant's growth season. For example, consider the scenario where the beginning and end of the growing season shift in parallel (Figure 2a). Such a situation may arise in temperate environments if the growth season of the focal plant species starts early in the year and as soon as temperatures allow it, shortly after snowmelt and say, and ends due to competition with other plants in which growth rates are also dependent on temperature. Especially, if the growth season of the focal plant is relatively short, the start and end of the season are likely to shift in parallel due to autocorrelation in temperatures. As another example, consider the scenario where the length of the growth season varies, at the same time as longer seasons reduce the productivity (Figure 2d). This situation can potentially occur in dry environments where the growth season of the focal plant species starts at the onset of rain and ends due to lack of soil water. While higher temperatures may increase plant growth rates they may also shorten the growth season since soil water then will be lost at a faster rate (cf. Chauhan et al., 2019).

When the total temperature sum over the growing season varies between years (i.e., variable *P*_tot_ in Equation 7) we predict that flowering according to a temperature sum is not the optimal strategy. OCR and TSR even show diametrically different responses to changes in *P*_tot_ (Figure 2c,e). This observation indicates under which kind of environmental variation when alternative strategies, such as photoperiod control, could have the upper hand over TSR. Consider the scenarios with changed season length (Figure 2d) and the scenario with variation in *P*_tot_ combined with unchanged start (*B*) and end (*E*) of the growing season (Figure 2e). A plant which flowers according to photoperiodic cues and thus has a fixed date for the onset of reproduction would in those two cases have a flowering time in between the OCR and the TSR. Even though photoperiod control would not coincide with the OCR, it would be closer to the optimum and thus be a better strategy than a TSR under such variation. This is an interesting observation in a climate change perspective, as it has been suggested that the plants that best track temperature change would be at an advantage (e.g., Willis, Ruhfel, Primack, Miller‐Rushing, & Davis, 2008). Note, however, that we still predict that photoperiodic control of flowering time would be inferior to TSR in the scenarios with an advanced season and other scenarios with unchanged *P*_tot_ (Figure 2a,b,d). Future research could test our model predictions by comparing whether plants with temperature or photoperiod control are more common in different biogeographic areas depending on the local climate with special attention to typical patterns of interannual variation in temperatures.

Apart from a constant *P*_tot_, the correspondence between TSR and OCR also requires a linear relationship between relative growth rate and temperature (Equation 1). This assumption underpins TSR method and has been supported in a meta‐analysis (Bonhomme, 2000). However, since high temperatures are detrimental to productivity, nonlinear models (Figure 1c) are more realistic in general. Our predictions, and those of temperature sum rules in general are thus probably more applicable to temperate areas with moderate temperature fluctuations than to environments where temperature variation is high (variation between *T*_2_ and *T*_3_ in Figure 1c, Schenk, 1996).

The annual plant growth model used here has been extended in many ways to represent more complicated life histories and processes affecting risks and growth rates along the season (Iwasa, 2000). Our analysis (Appendix 1) indicated that many of our conclusions may be carried over also to scenarios where growth is not only limited by temperature but in addition also by other additional factors such as herbivory and self‐shading. Other model versions include storage organs, roots, or consider perennial as opposed to annual life cycles (see Iwasa, 2000 for a review) or are adapted to annual social insects (e.g., Macevicz & Oster, 1976). For future studies, it can be noted that several properties for the basic model can be transferred to perennial plants (Iwasa & Cohen, 1989; Johansson et al., 2013). Our study thus provides an interesting connection between temperature control and timing of reproduction in annual plants as well as an inroad to link temperature control more generally to life history theory.

## CONFLICT OF INTEREST

None declared.

## AUTHOR CONTRIBUTIONS

The authors jointly conceived the study. JJ designed and analyzed the model and wrote the first draft of the manuscript. Both authors discussed the results and implications and contributed substantially to revisions.

## Figures and Tables

**Figure A1:**
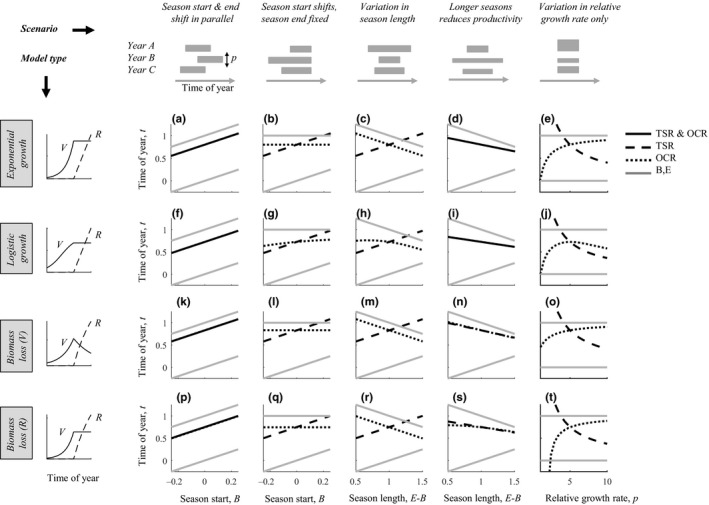
Effects of plant growth pattern on optimal flowering times compared to a strategy of flowering according to temperature sums. Five different scenarios of variation in seasonal growth conditions are considered, as illustrated in the top row. It is assumed that productivity remains constant over the season (but varies across years in the two leftmost scenarios). Each figure panel (a–t) shows how flowering time varies with environmental variation depending on whether the plant uses an optimal control rule (OCR) or a temperature sum rule (TSR). Plants grow exponentially (a–e, Equation 1), logistically (f–j, Equations A1 and A2) or according to a model version (Equations A3 and A4) where either vegetative (k–o) or reproductive (p–t) biomass is lost, simulating, for example, effects of herbivory. Baseline parameters: *p* = 5 and *V*_0_ = 1, *B* = 0, *E* = 1 for all models, *V*_max_ = 100 for the logistic growth model (panels f–j) and in the models accounting for biomass loss *m* = 1, *n* = 0 (panels k–o) and *m* = 0, *n* = 1 (panels p–t), respectively. In all cases, it is assumed that the TSR coincides with the OCR for the baseline parameters

## Data Availability

The manuscript contains no data to be archived.

## ENDNOTE

1
$\int_{B}^{F}p\left(t\right)dt=P↔\int_{B}^{F}p\left(t\right)dt+\int_{F}^{E}p\left(t\right)dt=P+\int_{F}^{E}p\left(t\right)dt↔$. $P_{\text{tot}}=P+\int_{F}^{E}p\left(t\right)dt↔P_{\text{tot}}-P=\int_{F}^{E}p\left(t\right)dt\int_{F}^{E}p\left(t\right)dt↔1=\int_{F}^{E}p\left(t\right)dt$. For the second step, we used Equation 6 and for the last step we used that for the optimal flowering time *P* = *P*_tot_ − 1 (Equation 7).

## APPENDIX 1

### Plant growth models with nonlinear relationships between relative growth rate and productivity

The basic plant growth model described by Equations 1 and 2 in the main text has been extended in several ways (see e.g., Iwasa 2000). In order to shed light on the generality of our findings, and as a robustness test, we shall compare our results with two additional model versions.

Firstly, we consider the fact that growth rates may slow down with the size of the developing plant, for example, due to self‐shading or competition within dense stands of conspecific plants. Following Deng et al. (2012) and Lindh et al. (2016) we consider that the plant grows logistically according to:

(A1) $$dV/dt=u\left(t\right)p\left(t\right)V\left(t\right)\left(1-V\left(t\right)/V_{\max}\right)\;\text{with}\;V\left(B\right)=V_{0}.$$

(A2) $$dR/dt=\left(1-u\left(t\right)\right)p\left(t\right)V\left(t\right)\left(1-V\left(t\right)/V_{\max}\right)\;\text{with}\;R\left(B\right)=0.$$

where *V*_max_ represents the maximum size of the plant and the remaining parameters as defined above.

Secondly, we consider that plant biomass may be lost due to, for example, senescence or herbivory. For this purpose, we follow King and Roughgarden (1983) (see also Macevicz & Oster, 1976)

(A3) $$dV/dt=u\left(t\right)p\left(t\right)V\left(t\right)\left(1-V\left(t\right)/V_{\max}\right)\;\text{with}\;V\left(B\right)=V_{0}.$$

(A4) $$dR/dt=\left(1-u\left(t\right)\right)p\left(t\right)V\left(t\right)\left(1-V\left(t\right)/V_{\max}\right)\;\text{with}\;R\left(B\right)=0.$$

These models have also been considered in the study of annual social insects (e.g., Macevicz & Oster, 1976; Poitrineau et al., 2009) for an application to annual social insects).

### Comparison of linear and nonlinear plant growth models

We compared how OCR and TSR respond to variation of *p*(*t*) depends on variation in the assumptions of the plant growth model. We consider different scenarios of between‐year variation caused by variation in the beginning (*B*) and end (*E*) of the season and relative growth rate (*p*) under the simplifying assumption that relative growth rate *p*(*t*) is constant (with *p*(*t*) = *p*) within the season (Figure A1, top). In all scenarios, we vary the parameters *B*, *E* and/or *p* around a set of baseline parameters (see legend of Figure A1 for details). We further assume that the timescale is measured in a way such that the length of the season is one in the scenario described by the baseline parameters (*B* = 0 and *E* = 1).

The flowering time of a plant following a temperature sum rule (TSR) is assumed to coincide with the optimal flowering time (given by Equation 6) for the baseline parameters. Specifically, we then assume that a plant which follows TSR initiates reproduction when *P*_tot_ − 1 = *pF* (cf. Equation 6). Since the length of the season is simply one, we further have that *P*_tot_ = *pE* = p and hence for a plant following TSR we have that *F* = *E* − 1/*p* = 1 − 1/*p*. In biological words, this simply means that increasing the temperature (which is proportional to relative growth rate *p*) causes the plant to flower earlier.

We start by comparing the basic growth model (Equations 1 and 2) with the logistic plant growth model (Equations A1 and A2). Since the basic growth model results in exponential growth when *p*(*t*) is constant we shall refer to the plant growth patterns produced by these different models as exponential and logistic growth, respectively.

Overall, the effect of changing from exponential (Figure A1a–e) to logistic growth (Figure A1f–j) has only relatively subtle effects on our predictions. With an exponential growth pattern OCR and TSR coincide with each other when the whole growth season is shifted (Figure A1a, where beginning and end of the season shift in parallel) and in a scenario where we vary the season length but at the same time keep *P*_tot_ constant (Figure A1d). With a logistic growth pattern, OCR and TSR coincide with each other in these two scenarios as well (Figure A1f,i). Hence, the principle we showed above (e.g., Figure 2a,b,d) that TSR and OCR coincide with each other whenever *P*_tot_ is constant, regardless of shape of *p*(*t*) or when the growth season start or ends, holds also when the growth pattern is logistic. In other scenarios (Figure A1b,c,e,g,h,j), *P*_tot_ is not constant between years and regardless of whether the plant grows exponentially or logistically, this causes TSR to deviate from OCR.

The optimal timing for switching to reproduction in the model version which accounts for biomass loss (Equations A3 and A4) is not affected by the rate of biomass loss if vegetative and reproductive biomass are lost at the same rate (i.e., if *m* = *n* in Equations A3 and A4, see e.g., Lindh et al., 2018; Macevicz & Oster, 1976). Hence, in this case, TSR and OCR will coincide under exactly the same circumstances as predicted by the basic growth model (Equations 1 and 2). It is, however, also conceivable that vegetative and reproductive biomass are lost at different rates, for example, if herbivory mainly reduces vegetative biomass (*m* > *n*) because reproductive biomass, such as seeds, is strongly protected from consumption or if seed predation causes large loss of reproductive biomass without affecting vegetative biomass (*n* < *m*). Our numerical simulations show that unequal rates of biomass loss may influence how the optimal timing of reproduction depends on the relative growth rate *p* (Figure A1o,t) but when we compare TSR and OCR in the different scenarios considered in Figure A1, we obtain qualitatively similar results (Figure A1k–t) to when we use the basic growth model (Figure A1a–e). When vegetative and reproductive biomass is lost at unequal rates TSR and OCR do not coincide exactly with each other when this is predicted by the basic model (Figure A1a,d,k,n,p,s) but then the difference between TSR and OCR is very small (<0.05).
